# Enlarged cardiophrenic lymph nodes predict disease involvement of the upper abdomen and the outcome of primary surgical debulking in advanced ovarian cancer

**DOI:** 10.1111/aogs.13835

**Published:** 2020-03-18

**Authors:** Anna K. Luger, Fabian Steinkohl, Friedrich Aigner, Werner Jaschke, Christian Marth, Alain G. Zeimet, Daniel Reimer

**Affiliations:** ^1^ Department of Radiology Innsbruck Medical University Innsbruck Austria; ^2^ Department of Obstetrics and Gynecology Innsbruck Medical University Innsbruck Austria

**Keywords:** cardiophrenic lymph node, ovarian cancer, predictive value, prognostic impact, residual disease

## Abstract

**Introduction:**

The outcome of ovarian cancer patients is highly dependent on the success of primary debulking surgery in terms of postoperative residual disease. This study critically evaluates the clinical impact of preoperative radiologic assessment of the cardiophrenic lymph node (CPLN) status in advanced ovarian cancer.

**Material and methods:**

Baseline CT scans of 178 stage III and IV ovarian cancer patients were retrospectively reviewed by two independent radiologists. CPLN enlargement defined at a short‐axis ≥5 mm was evaluated for its prognostic value and predictive power of upper abdominal tumor involvement and the chance of complete intra‐abdominal tumor resection at primary debulking surgery. Only patients without surgically removed CPLN were eligible for this study.

**Results:**

Enlarged CPLNs were detected in 50% of patients and correlated with radiologically suspicious (*P* = .028) and histologically confirmed (*P* = .001) paraaortic lymph node metastases. CPLNs ≥ 5 mm were associated with high CA‐125 levels at baseline and revealed independent prognostic relevance for progression‐free survival (hazard ratio [HR] 2.14, 95% confidence interval [CI] 1.33‐3.42) and overall survival (HR 2.18, 95% CI 1.16‐4.08). Noteworthy, patients with enlarged CPLNs nonetheless benefit from complete intra‐abdominal tumor debulking in terms of an improvement in progression‐free survival (HR 0.60, 95% CI 0.38‐0.94) and overall survival (HR 0.59, 95% CI 0.35‐0.82). Enlarged CPLNs correctly predicted carcinomatosis of the upper abdomen in 94.6%. A predictive score of complete tumor debulking, termed CD‐score, which integrates, beside a CPLN short axis <5 mm, an ascites volume <500 mL, and CA‐125 levels <500 U/mL at baseline, correctly predicted complete intra‐abdominal debulking in 100% of patients.

**Conclusions:**

CPLNs ≥5 mm predict upper abdominal tumor involvement. The application of the CD‐score predicted complete macroscopic tumor resection at primary surgery in all of the patients. Although, CPLN pathology suggests extra‐abdominal disease, we consistently demonstrated that patients nonetheless benefit from complete intra‐abdominal tumor resection.

**Abbreviations**
CD‐score: complete tumor debulking score
CI: confidence interval
CPLN: cardiophrenic lymph node
FIGO: International Federation of Gynecology and Obstetrics
HR: hazard ratio
IQR: interquartile range

**Key message**

Preoperative radiologic assessment of cardiophrenic lymph nodes predicts upper abdominal tumor involvement and the chance of complete surgical debulking. Even though enlarged cardiophrenic lymph nodes remain surgically unremoved, complete intra‐abdominal tumor resection translates into significant survival benefit.

## 1 INTRODUCTION

Ovarian cancer is the most lethal gynecologic malignancy in western countries.^1^ Mainly diagnosed at an advanced stage, prognosis is fundamentally influenced by residual disease after debulking surgery.^2^ The achievement of optimal cytoreduction is highly influenced by the extent of carcinomatosis, whereby tumor spread in the upper abdomen is a major obstacle to complete macroscopic tumor resection.^3^ In this context, high‐resolution imaging modalities are used in the preoperative evaluation of tumor spread in order to predict complete tumor resectability.^1^ Over the last decades, extensive pelvic and paraaortic lymphadenectomy up to the left renal vein has been performed to remove potentially cancer‐affected retroperitoneal lymph nodes. However, data from the LION trial did not show any benefit of systematic lymph node dissection in advanced stage ovarian cancer in the case of radiologic unsuspicious nodes.^4^ Despite the intention to remove occult cancer by performing systematic lymphadenectomies, virtually no attention has been paid to the frequently observed enlarged cardiophrenic lymph nodes (CPLNs).^5^, ^6^ Anatomically, the cardiophrenic region is a fat‐filled space between the mediastinum, heart base, diaphragm and chest wall. Small visible lymph nodes in this area can be physiological but there usually are fewer than two, each with a diameter of <5 mm.^7^ The most classical route of dissemination of epithelial ovarian cancer is the spread of exfoliated free‐floating cancer cells throughout the abdominal cavity via the physiological peritoneal fluid, leading to peritoneal carcinomatosis. The major portion of the lymph drainage to the CPLNs occur via sub‐peritoneal plexuses located adjacent to the diaphragm. Holloway et al found a correlation between peritoneal metastases and the enlargement of para‐cardiac lymph nodes.^6^ In RECIST 1.1, lymph nodes, independently of their location, are considered to be of pathologic relevance when their short‐axis is >10 mm.^8^ Nevertheless, several groups have demonstrated an adverse prognostic impact on survival of ovarian cancer patients exhibiting CPLNs with >5 mm short‐axis.^6^, ^9^, ^10^ Thus, the European Society of Urogenital Radiology (ESUR) has defined pathologic enlarged CPLNs at a cutoff a ≥5 mm short‐axis dimension.^11^

The study presented here aims to evaluate critically the prognostic impact of enlarged CPLNs in advanced stage ovarian neoplasms, especially considering the debulking outcome at primary surgery. In addition, we studied the impact of radiologically assessed pathologic CPLNs to predict carcinomatosis of the upper abdomen and complete macroscopic tumor resection at primary debulking surgery.

## 2 MATERIAL AND METHODS

### 2.1 Patient cohort

The study population consists of 178 patients with the International Federation of Gynecology and Obstetrics (FIGO) stage III/IV, invasive epithelial ovarian cancer diagnosed between 2000 and 2016 in our department. Patients receiving neoadjuvant chemotherapy followed by interval debulking surgery were excluded, and only individuals without surgically removed CPLN were investigated. From 356 patients eligible for the study, every second patient was chosen at random for further analyses (Figure S1). Median age at diagnoses was 64.6 years (interquartile range [IQR] 50.8‐72.7). Recurrence was observed in 66.9% (n = 119) of patients and the median progression‐free survival was 12.0 months (IQR 5.5‐30.5). Eighty patients (44.9%) died during a median time of follow up of 49.6 months (IQR 32.89‐66.26). Primary debulking surgery was performed in the entire population by dedicated teams including at least one certified gynecologic oncologist, and all patients received adjuvant platinum‐based chemotherapy. We defined “No residual disease” as complete macroscopic tumor resection at the end of debulking surgery. A systematic pelvic and paraaortic lymphadenectomy (removal of ≥20 retroperitoneal lymph nodes according to Panici et al12) was performed in 84.2% of patients and lymph node sampling in 4.5%. The median number of removed nodes was 26 (IQR 7‐37), and 68% (n = 88) of patients exhibited histologically proven retroperitoneal lymph node metastases. Clinicopathologic parameters are shown in Table 1.

**Table 1:** Patient characteristics (n = 178)

	CPLN negative		CPLN positive (≥5 mm)		*P* value^a^
	Median	IQR	Median	IQR	
Age (y)	64.70	50.15‐73.54	63.48	51.42‐71.42	.872
FIGO stage					
IIIA/IIIB	16	18.0	2	2.2	
IIIC	62	69.7	11	12.4	
IVA	11	12.3	2	2.2	
IVB	0	0	74	83.2	.0001
Histologic subtype					
High‐grade serous	69	77.5	73	82.0	
Low‐grade serous	12	13.5	3	3.4	
Mucinous	1	1.1	2	2.2	
Endometrioid	5	5.6	8	9.0	
Clear cell	2	2.3	3	3.4	.205
Grading					
G1	13	14.7	4	4.5	
G2	43	48.3	39	43.8	
G3	33	37.0	46	51.7	.029
Debulking surgery					
Yes	89	100	89	100	
No	0	0	0	0	
Residual disease					
No residual disease	80	89.9	53	59.6	
Residual disease	9	10.1	36	40.4	.0001
Lymphadenectomy^b^					
Systematic (≥20 nodes)	79	88.8	71	79.8	
Sampling (<20 nodes)	2	2.2	6	6.7	.199
Adjuvant chemotherapy					
Carboplatin + paclitaxel	77	86.5	73	82.0	
Carboplatin	11	12.4	13	14.6	
Cisplatin + endoxan	1	1.1	3	3.4	.529
Platinum response^c^					
Refractory + resistant	14	15.7	21	23.6	
Sensitive	75	84.3	68	76.4	.403
Recurrence					
Yes	42	47.2	77	86.5	
No	47	52.8	12	13.5	.0001
Death					
Yes	27	30.3	53	59.6	
No	62	69.7	36	40.4	.0001

Notes

Values are expressed as median, IQR for ‘Age’ and as n, % for all other characteristics.

Abbreviations: CPLN, cardiophrenic lymph node; FIGO, Fédération Internationale de Gynécologie Obstétrique; IQR, interquartile range.

^a^ Differences assessed by Mann‐Whitney *U* test (Age) or chi‐square test.

^b^ Systematic retroperitoneal lymphadenectomy.^12^

^c^ Platinum sensitivity according to The Fifth Ovarian Cancer Consensus Conference of the Gynecologic Cancer InterGroup: recurrent disease.^13^

### 2.2 Imaging review

Prior to treatment, all patients had baseline contrast enhanced CT scans using standard institutional protocols on multi‐detector row scanners (Somatom Sensation Cardiac 64, Somatom Sensation 16, and Somatom Plus 4 Volume Zoom [Siemens Medical Solutions, Forchheim, Germany]). Collimation was .6‐2.5 mm, 120 kVp, and 155‐280 mA, pitch of .88‐1.25; and reconstruction was performed at a slice thickness of ≤5 mm. Intravenous contrast agent (Iopromide [300 mg I/mL]; Ultravist 370 [Bayer HealthCare Pharmaceuticals, San Francisco, CA, USA]) was administered at a rate of 2‐3 mL/s.

All CT scans were centrally reviewed by two independent radiologists (A.L. and F.A.): The number, the location with respect to the heart (right, left or anterior) and the short‐axis dimension of the largest CPLN were measured in every single patient. In addition, the amount of ascites, retroperitoneal lymphadenopathy and the localization of intra‐abdominal carcinomatosis, especially focusing on upper abdominal involvement, were evaluated. Based on previous studies, we defined radiologically suspect diaphragmatic thickness at a cutoff >2 mm.^10^ In addition, the sites of recurrence were estimated by follow‐up CT scans in all patients at the time of their first relapse.

### 2.3 Statistical analyses

According to the European Society of Urogenital Radiology (ESUR) guidelines^11^ and a recently published study,^10^ we defined pathologically enlarged CPLNs at a cutoff ≥5 mm short‐axis dimension. All statistical analyses were performed using the SPSS® Statistics software version 24 (IBM, Armonk, NY, USA). Differences in survival were assessed using the Kaplan‐Meier method with log‐rank test and Cox's proportional hazard models for uni‐ and multivariate analyses. Association of CPLNs ≥5 mm and clinicopathological parameters was evaluated using Mann‐Whitney *U* or Chi‐square tests. Statistical significance was defined as *P* < .05.

### 2.4 Ethical approval

The utilization of patient data for study purposes was handled according to the World Medical Association Declaration of Helsinki 2008 and national legal norms. Institutional review board approval was granted by means of a general waiver for studies with retrospective data analysis (Ethics Committee, Innsbruck Medical University; 20 February 2009).

## 3 RESULTS

### 3.1 Radiological detection of cardiophrenic lymph nodes and clinicopathological parameters

In a cohort of 178 advanced ovarian cancer patients, preoperative CT scans revealed CPLNs of any size in 90.4% (n = 161) of patients; 50.0% (n = 89) of patients exhibited lymph nodes ≥5 mm in short‐axis. In 48.9%, the dominant lymph node was located at the right side of the cardiophrenic space, whereas in 24.2% or 17.4%, respectively, it was located near the midline or at the left side. A median of three (IQR 2‐5) enlarged CPLNs were detected. The number of enlarged nodes was not associated with clinicopathological parameters or patient outcome.

Conversely, CPLN with a short‐axis ≥5 mm was associated with radiologically suspicious paraaortic nodes (odds ratio 2.12, 95% confidence interval [CI] 1.06‐4.34; *P* = .028) and histologically confirmed paraaortic lymph node metastases (odds ratio 3.39, 95% CI 1.61‐7.16; *P* = .001). In addition, enlarged CPLNs were associated with a higher CA125 level at baseline (median value 725 U/mL, IQR 256‐2154) as compared with unsuspicious CPLNs (323 U/mL, IQR 100‐1056; *P* = .0001). We found no association between enlarged CPLNs and patient age at diagnosis or response to adjuvant chemotherapy. Neither a pre‐therapeutic, CT‐estimated ascites volume >500 mL nor radiologically evident pleural effusion (n = 26) was associated with pathologically enlarged CPLNs. Clinicopathological parameters according to the CPLN status are shown in Table 1.

Complete intra‐abdominal tumor resection was achieved in 133 patients. Eighty (60.2%) of those patients exhibited recurrence during follow up. The predominant site of recurrence was the intra‐abdominal cavity (87.5%), not the retroperitoneal space (8.8%) or an exclusive organ‐related metastatic spread (1.7%).

### 3.2 Prognostic impact of enlarged cardiophrenic lymph nodes

**Figure 1:**
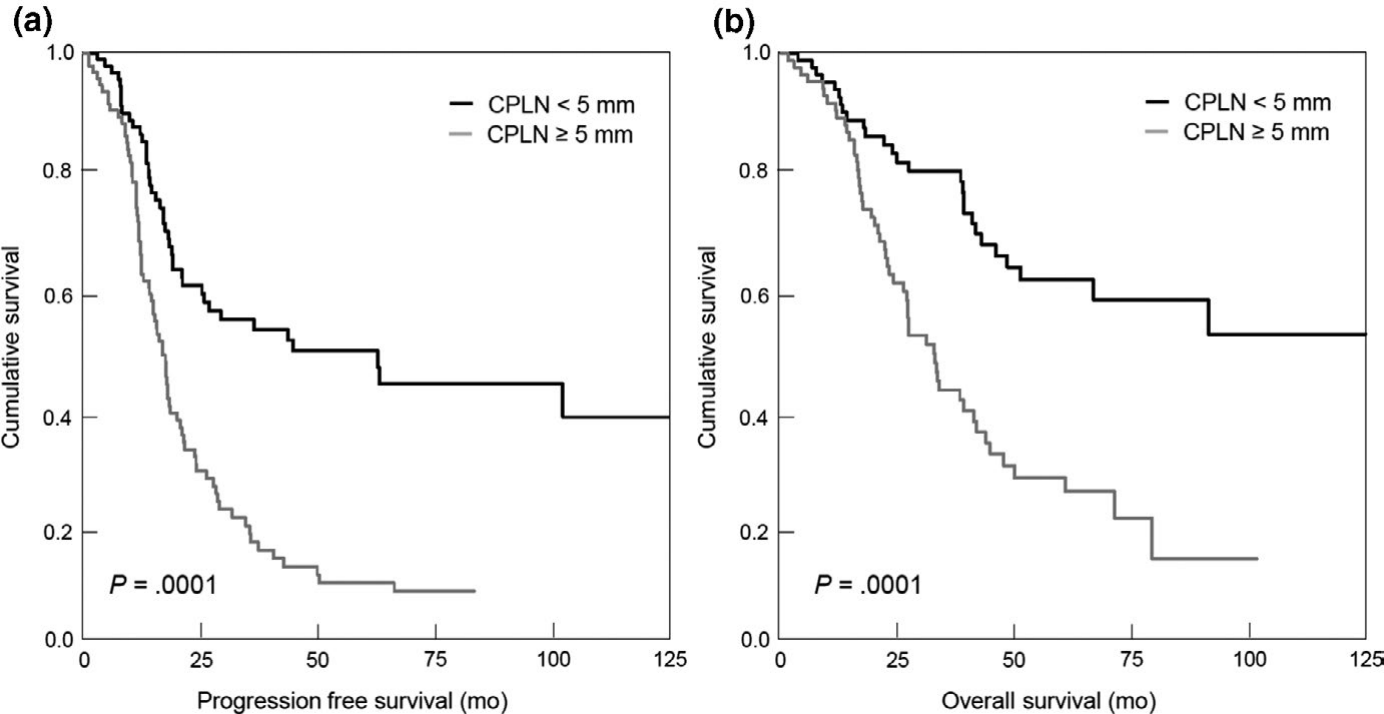
Progression‐free (A) and overall survival (B) of patients (n = 178) according to their cardiophrenic lymph node (CPLN) status (<5 mm vs ≥5 mm)

CPLNs ≥5 mm were associated with impaired progression‐free and overall survival (Figure 1A,B). In addition, the CPLN status proved to be of independent prognostic relevance in Cox's regression models including the age at diagnosis, residual disease after debulking surgery, CA125 level, FIGO stage and retroperitoneal lymph node positivity as covariates (Table 2). Stratification of patients based on the presence of positive retroperitoneal nodes did not change the prognostic strength of enlarged CPLNs (Figure S2).

**Table 2:** Multivariate Cox regression model

	Progression‐free survival			Overall survival		
	HR	95% CI	*P* value	HR	95% CI	*P* value
Age (y)						
>64.6	1.48	.92‐2.39	.109	1.96	1.05‐3.66	.035
CA‐125						
≥35 U/mL	1.89	.68‐5.23	.222	3.89	0.53‐9.99	.183
Paraaortic nodes^a^						
Positive	2.19	1.32‐3.64	.002	1.69	0.83‐3.43	.147
FIGO stage						
FIGO IV^b^	0.68	.33‐1.39	.286	0.86	0.37‐1.99	.719
Residual disease						
Yes	2.44	1.23‐4.84	.011	2.17	1.11‐4.69	.028
CPLN dimension						
≥5 mm	2.92	1.38‐6.16	.005	2.43	1.12‐5.81	.019

Abbreviations: CI, confidence interval; CPLN, cardiophrenic lymph node; HR, hazard ratio.

^a^ Histologically positive paraaortic lymph nodes.

^b^ FIGO IIIA‐IIIC vs FIGO IVA and IVB.

When patients were adjusted for their outcome of debulking surgery, enlarged CPLNs retained prognostic relevance only in the patients with macroscopically complete tumor resection (progression‐free survival: hazard ratio [HR] 2.02, 95% CI 1.14‐3.55; *P* = .015; overall survival: HR 2.46, IQR 1.54‐3.93; *P* = .0001) (Figure S3). No significant impact was revealed in the subgroup with a macroscopic tumor residual (Figure S4).

**Figure 2:**
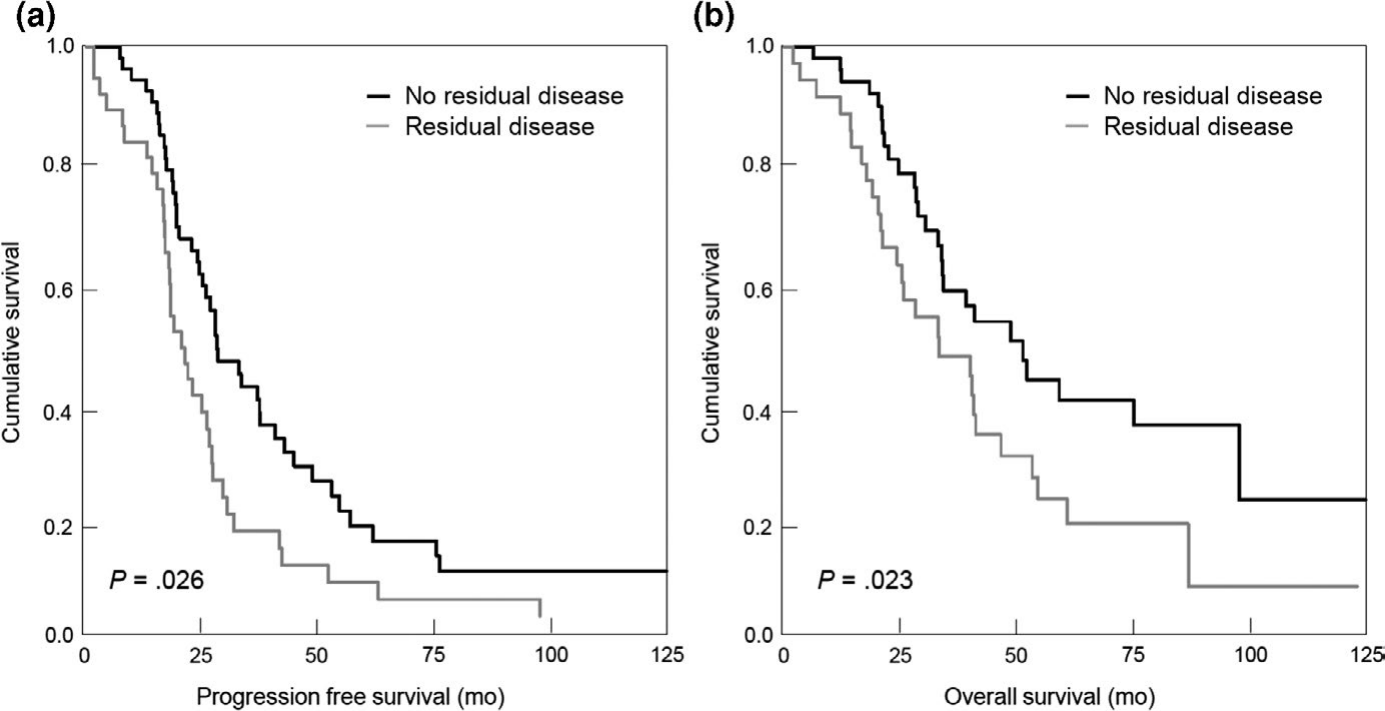
Progression‐free (A) and overall survival (B) in the subgroup of patients with enlarged cardiophrenic lymph nodes (n = 89) according to residual disease after primary debulking surgery (No residual disease vs Residual disease)

We next evaluated whether patients with enlarged CPLNs would still benefit from complete intra‐abdominal tumor debulking. In fact, patients exhibiting a pathological CPLN status (n = 89) benefit from complete intra‐abdominal tumor resection in terms of an improvement in progression‐free survival of 4.8 months (HR 0.60, 95% CI 0.38‐0.94; *P* = .027) and in overall survival of 14.4 months (HR 0.59, 95% CI 0.35‐0.82; *P* = .030) (Figure 2A,B).

### 3.3 Predictive value of enlarged cardiophrenic lymph nodes for upper abdominal disease

Intraperitoneal carcinomatosis was radiologically evident in 84.8% (n = 151) of patients, and 40.9% (n = 72) had a radiologic diagnosis of upper abdominal spread. Enlarged CPLNs showed high concordance with radiologically diagnosed carcinomatosis of the upper abdomen (*P* = .0001). However, intraoperative detected carcinomatosis of the upper abdomen was radiologically not evident in 35.1% of patients. Most of those misdiagnosed cases (24/26) exhibited miliary tumor spread. Correlation of enlarged CPLNs with intraoperatively confirmed upper abdominal disease yielded sensitivity and specificity rates of 94.6% (70/74) and 83.0% (83/100), respectively (positive predictive value 80.5%, negative predictive value 95.4%). Even in the patients exhibiting miliary upper abdominal spread, CPLN enlargement was able to predict carcinomatosis of the upper abdomen in 92.7%.

### 3.4 Predictive value of enlarged cardiophrenic lymph nodes for complete tumor resection

Macroscopic complete surgical tumor resection was achieved in 89.9% (80/89) of the patients without enlarged CPLNs. Nevertheless, in our hands, complete intra‐abdominal tumor debulking was also achieved in 53 of 89 patients (59.5%) exhibiting CPLNs ≥5 mm, translating into a low negative predictive value of 40.4% (Table 3). To improve the prediction of optimal tumor debulking, we integrated the CA‐125 level at primary diagnosis, the estimated ascites volume in baseline CT scans, and the CPLN status into a clinical score of complete tumor debulking (CD‐score). Individual parameters or a combination of two did not markedly improve the test performance. Only the integration of all three parameters led to a substantial improvement of prediction. In fact, patients fulfilling all the three criteria, namely, (1) a CA‐125 level ≤500 U/L, (2) a radiologically estimated ascites volume <500 mL and (3) a CPLN <5 mm, had a chance of complete tumor resection in 100% (Table 3).

**Table 3:** Predictive value for complete tumor debulking of CPLN status in different scores

	Sensitivity (%)	Specificity (%)	PPV (%)	Negative predictive value (%)
CPLN <5 mm	60.2	80.0	89.9	40.4
Ascites <500 mL	31.6	93.3	93.3	31.5
CA‐125 ≤500 U/mL	58.6	60.0	81.3	32.9
CA‐125 ≤500 U/mL + Ascites <500 mL	39.5	86.2	88.2	35.2
CPLN < 5 mm + Ascites <500 mL	40.3	100.0	93.1	45.2
CPLN < 5 mm + CA‐125 ≤500 U/mL	66.2	76.5	86.4	50.0
CPLN <5 mm + Ascites <500 mL + CA‐125 ≤500 U/mL (CD‐score)	87.2	100.0	100.0	86.8

Abbreviations: CPLN, cardiophrenic lymph node; CD‐score, complete tumor debulking at upfront surgery score; NPV, negative predictive value; PPV, positive predictive value.

## 4 DISCUSSION

In the cohort examined here, we found enlarged CPLNs (short‐axis ≥5 mm) in 50% of patients. In the literature, detection rates of between 11% and 62% are described depending on the discriminatory diameter used and the patient cohort investigated.^6^, ^9^, ^10^, ^11^, ^14^, ^15^, ^16^, ^17^ A stepwise increase of radiologically used CPLN short‐axis from ≥3 to ≥10 mm yields to a substantial decrease in the detection rate of potentially pathological CPLNs.^14^ This effect is corroborated by several studies and was confirmed by our own data (Table S1). The histological confirmation rate of enlarged CPLNs ranges between 85% and 95%^10^, ^17^, ^18^, ^19^, ^22^ and is obviously independent of different radiological short‐axis diameters (Table S1). Thus, a noncritical increase of the radiologically applied CPLN short‐axis bears a risk of underdiagnosis of tumor‐affected lymph nodes. Therefore, and in line with the ESUR guidelines,^11^ we opted for a radiologic cutoff of a CPLN short‐axis ≥5 mm. The preoperatively radiological detection of enlarged and putatively cancer‐affected CPLNs has not been routinely integrated into ovarian cancer staging and treatment considerations so far. Integration of enlarged CPLNs, which may reflect extra‐abdominal disease, would implicate a stage shift from FIGO III and IVA to FIGO IVB of 38.2% in our cohort. As shown in Table S1, FIGO stage shift is highly dependent on the radiological short‐axis cutoff used. All this is raising the question of whether a cancer‐affected CPLN is really of clinical impact, or the current FIGO classification is lacking in precision in defining FIGO IVB disease with clinical relevance. A similar debate has already been opened concerning the impact of abdominal wall metastases^20^ and inguinal nodes.^21^

Regarding the clinical impact of radiologically enlarged CPLNs in ovarian cancer, several studies have shown that enlarged CPLNs are associated with worse patient outcome.^6^, ^10^, ^14^, ^15^, ^16^ Our data corroborate this finding by demonstrating a significant impairment in survival in advanced stage ovarian cancer patients with radiologically detected enlarged CPLNs. The fact that enlarged CPLNs cause a deterioration in survival despite complete intra‐abdominal tumor resection further highlights the clinical impact of CPLN in ovarian cancer. However, Prader et al^10^ recently demonstrated that a removal of radiologically enlarged CPLNs did not confer a significant survival benefit compared with a matched control population without resection of enlarged CPLN. Noteworthy, 84.6% of the removed and enlarged CPLNs in this study were revealed to be histologically tumor‐involved. In our study cohort, nearly all recurrences occurred predominately within the abdominal cavity and not in the retroperitoneal lymph nodes. All this indicates that in ovarian cancer the intra‐abdominal tumor spread has another, probably more important pathological weight compared with the lymphatic spread in the retroperitoneal space. This assumption is also corroborated by the results of the LION study, where in advanced stage ovarian cancer patients a systematic pelvic and paraaortic lymph node dissection did not improve survival outcome, although occult lymph node metastases were detectable in 55.7% of patients.^4^ Consequently, it seems that for successful therapy a complete intra‐abdominal tumor resection has a higher clinical impact than the removal of cancer cells scattered in the lymph nodes. In line with that, we found that patients exhibiting enlarged CPLNs, nevertheless benefitted from complete tumor debulking. In this context, du Bois et al^2^ demonstrated improved survival in optimally debulked patients with pleural affections.

Notwithstanding, the appearance of enlarged CPLNs remained an independent prognostic parameter in this retrospective study. As removal of those nodes does not seem to be of therapeutic value, it may be that CPLN involvement represents a surrogate parameter of extensive tumor load, especially in upper abdomen. We demonstrate here that patients exhibiting enlarged CPLNs had a higher likelihood of upper abdominal tumor involvement, which is in agreement with data published by Prader et al.^10^ Upper abdominal disease is often associated with a high Sugarbaker Peritoneal Carcinomatosis Index (PCI) and represents a main obstacle to macroscopically complete tumor resection.^3^, ^24^ Imaging‐only based prediction has been shown to be ineffective, since miliary carcinomatosis is often missed by radiology.^25^, ^26^ In our collective, upper abdominal carcinomatosis remained preoperatively undetected in 35.1%, most of those cases small volume miliary spread being discovered during surgery. Even in that subgroup, CPLNs ≥5 mm predicted carcinomatosis correctly in the upper abdomen in 93%. Therefore, a preoperative evaluation of cardiophrenic lymphadenopathy may represent a helpful tool for initial treatment planning, including the transfer of patients to high‐volume centers. Institutions specialized in upper abdominal surgery are often able to achieve complete tumor resection despite extensive upper abdominal disease. In our collective, complete debulking in the case of CPLN enlargement and upper abdominal tumor involvement was feasible in approximately two‐thirds of patients.

Several studies including the recently published paper by Prader et al^9^, ^10^, ^16^ have demonstrated an inverse impact of enlarged CPLNs on complete resection rates. Beyond this, however, our study focuses on a systematic evaluation of the predictive value of CPLN enlargement in combination with other clinicopathological parameters. Prediction of debulking outcome based on radiologically enlarged CPLN only, turned out to be unreliable in the present study. Referring to the AGO‐DESKTOP score for debulking outcome in recurrent ovarian cancer,^27^, ^28^ we applied a score to predict complete tumor debulking (CD‐score) during upfront surgery by combining the volume of ascites, CA‐125 level at baseline, and the radiological CPLN status. In our cohort, complete intra‐abdominal tumor resection was achieved in all of the patients who exhibited all three parameters below their defined threshold values. When CPLN short‐axis <5 mm was considered alone, complete resectability was achieved in 90% of patients. This was associated with a low negative predictive value of 40%. The application of the CD‐score resulted in a substantial increase of the negative predictive value to 87%. However, we did not take into account other factors, especially the molecular subtype of an individual cancer, which may greatly influence dissemination pattern, invasion efficiency, and thus resectability. In line with this, data from a recent study underline the pivotal role of distinct epithelial‐mesenchymal transition (EMT) gene signatures in the formation of different (miliary vs non‐miliary) dissemination patterns in high‐grade serous ovarian cancers.^30^ Needless to say, our CD‐score needs to be validated in a larger multicenter study before being introduced into clinical practice.

Our study has several limitations. It was retrospectively designed; however, to minimize selection bias, a large number of consecutive patients over a period of 16.5 years were randomly selected. As in none of the study patients were radiologically enlarged CPLNs surgically removed, we are not able to prove a frank metastatic lymph node involvement. The high association between radiologic enlarged CPLNs and histologically proven cancer‐affected paraaortic lymph nodes could argue in favor of a tumor involvement in the majority of enlarged nodes. In addition, previous studies have shown that in radiologically enlarged CPLNs histologic tumor spread was demonstrated in 85%‐95%.^10^, ^17^, ^18^, ^19^, ^22^ Nonetheless, the possibility of a reactive lymph node enlargement based on “work hypertrophy” in the case of ascites has to be kept in mind.^9^ Regarding the latter concern, our data did not reveal a positive association between enlarged CPLNs and the amount of ascites, or the occurrence of pleural effusions.

## 5 CONCLUSION

The data presented here of an inverse association of enlarged CPLN with complete resection rates and patient survival confirm the recently published data by Prader et al.^10^ In addition, we demonstrate here that a preoperative radiological evaluation of the CPLN status anticipates tumor involvement of the upper abdomen, which is especially useful in those patients with miliary carcinomatosis missed by conventional imaging. Beyond this, we provide for the first time an easy‐to‐perform clinical score to predict the chance of complete surgical debulking, which could help clinicians in tailoring adequate therapy strategies in ovarian cancer. In addition, we demonstrate that patients with enlarged CPLNs nonetheless benefit significantly from all possible surgical efforts translating into a complete intra‐abdominal tumor resection.

## CONFLICT OF INTEREST

The authors have stated explicitly that there is no conflict of interest in connection with this article.

## Supporting information


**Figure S1**

**Figure S2**

**Figure S3**

**Figure S4**

**Table S1**
