# Supplementary figures and images for: Enlarged cardiophrenic lymph nodes predict disease involvement of the upper abdomen and the outcome of primary surgical debulking in advanced ovarian cancer
Source: Acta Obstet Gynecol Scand. 2020 Mar 18;99(8):1092–9. doi: 10.1111/aogs.13835 (PMC7496971; doi:10.1111/aogs.13835)

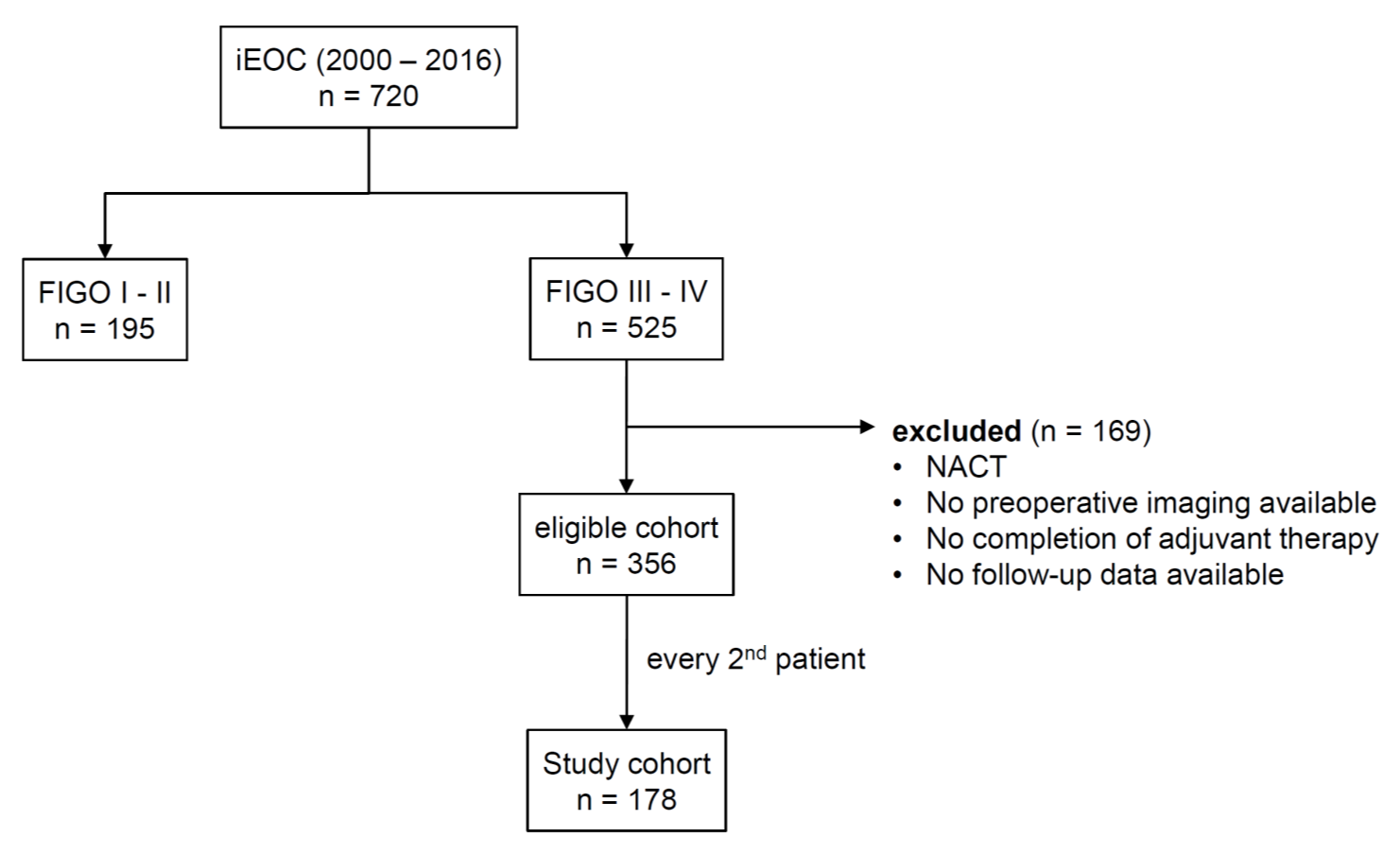

Supplement: Supplementary file 1 — Figure S1 [file AOGS-99-1092-s001.tif]

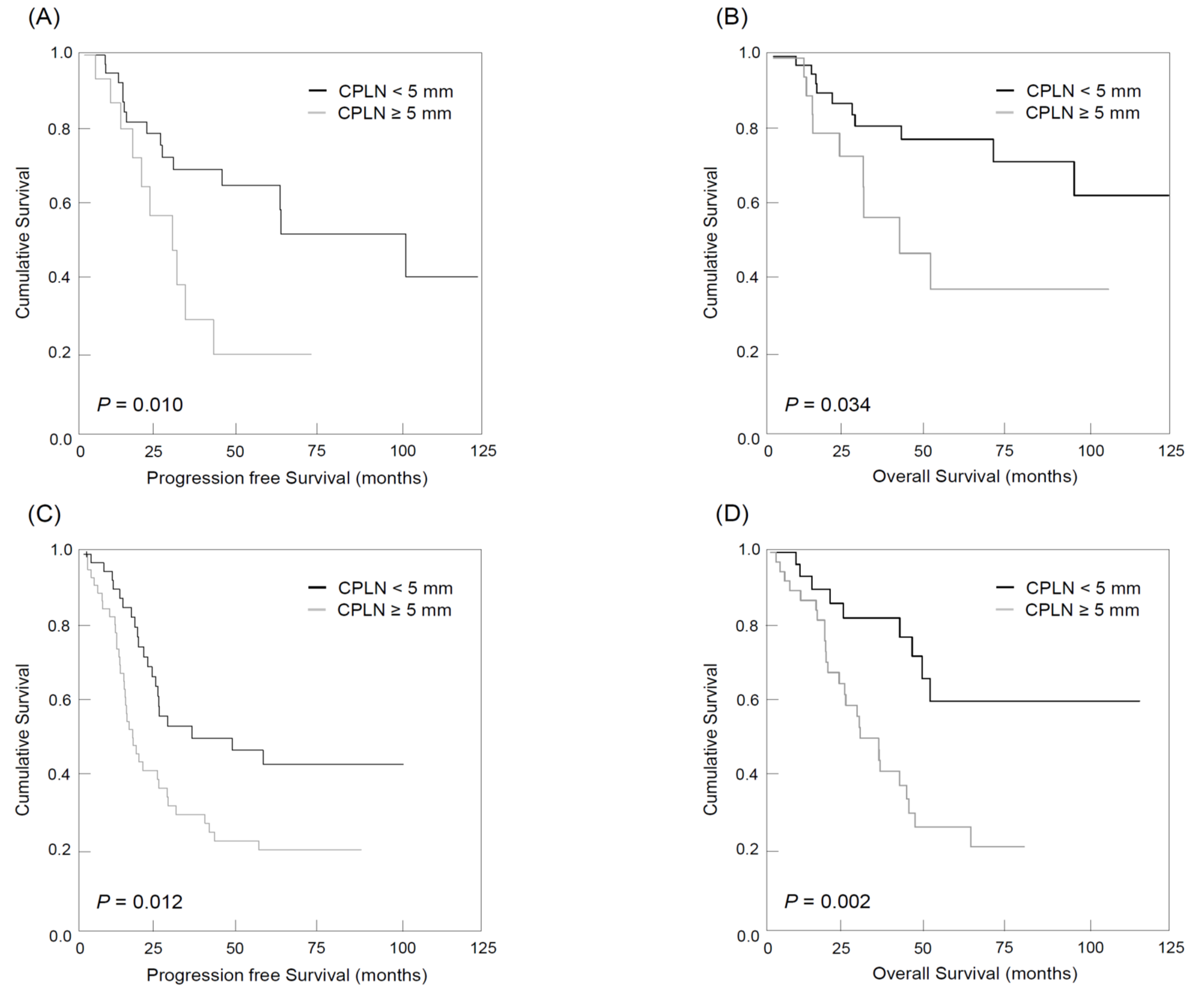

Supplement: Supplementary file 2 — Figure S2 [file AOGS-99-1092-s002.tif]

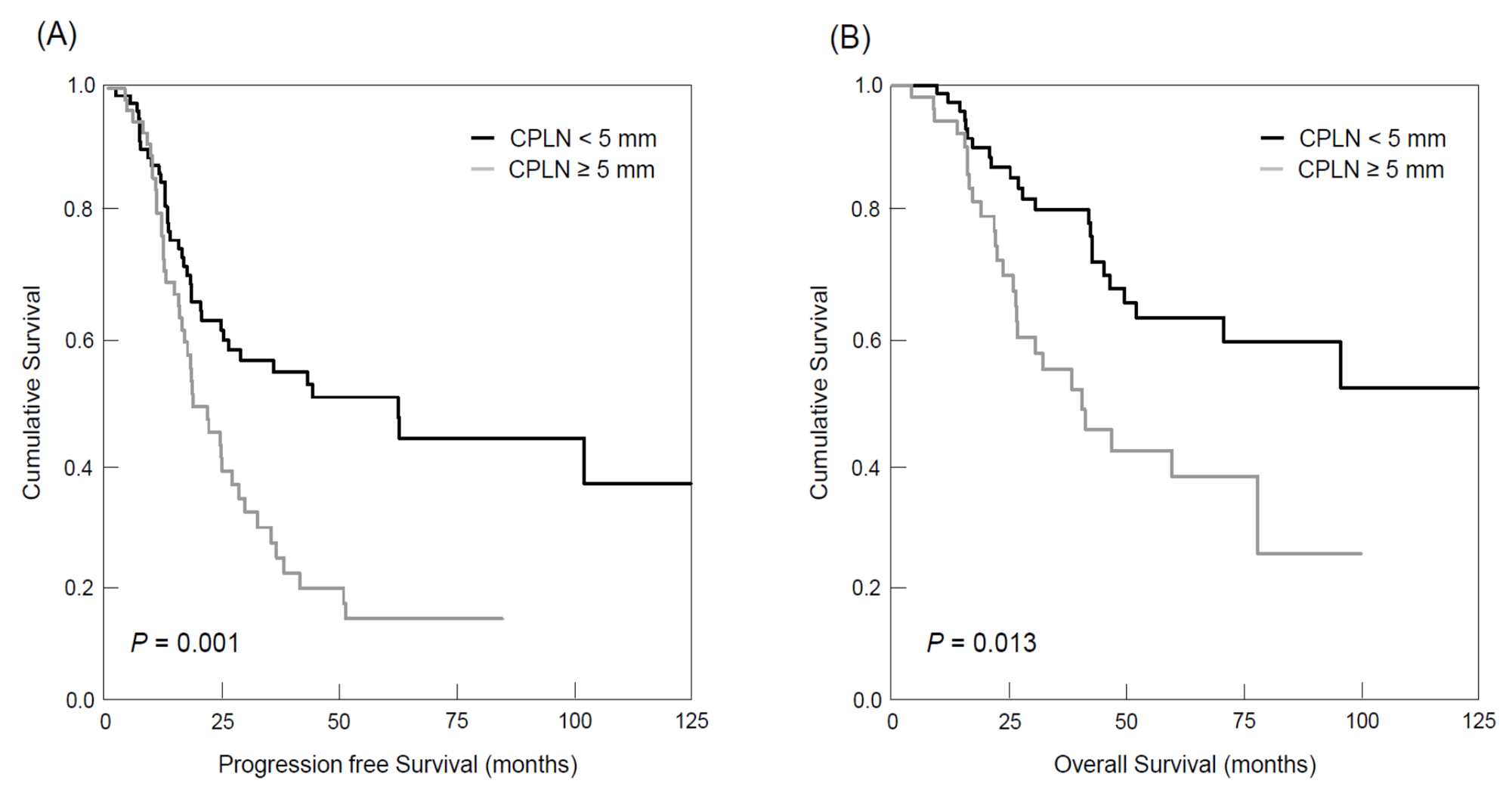

Supplement: Supplementary file 3 — Figure S3 [file AOGS-99-1092-s003.tif]

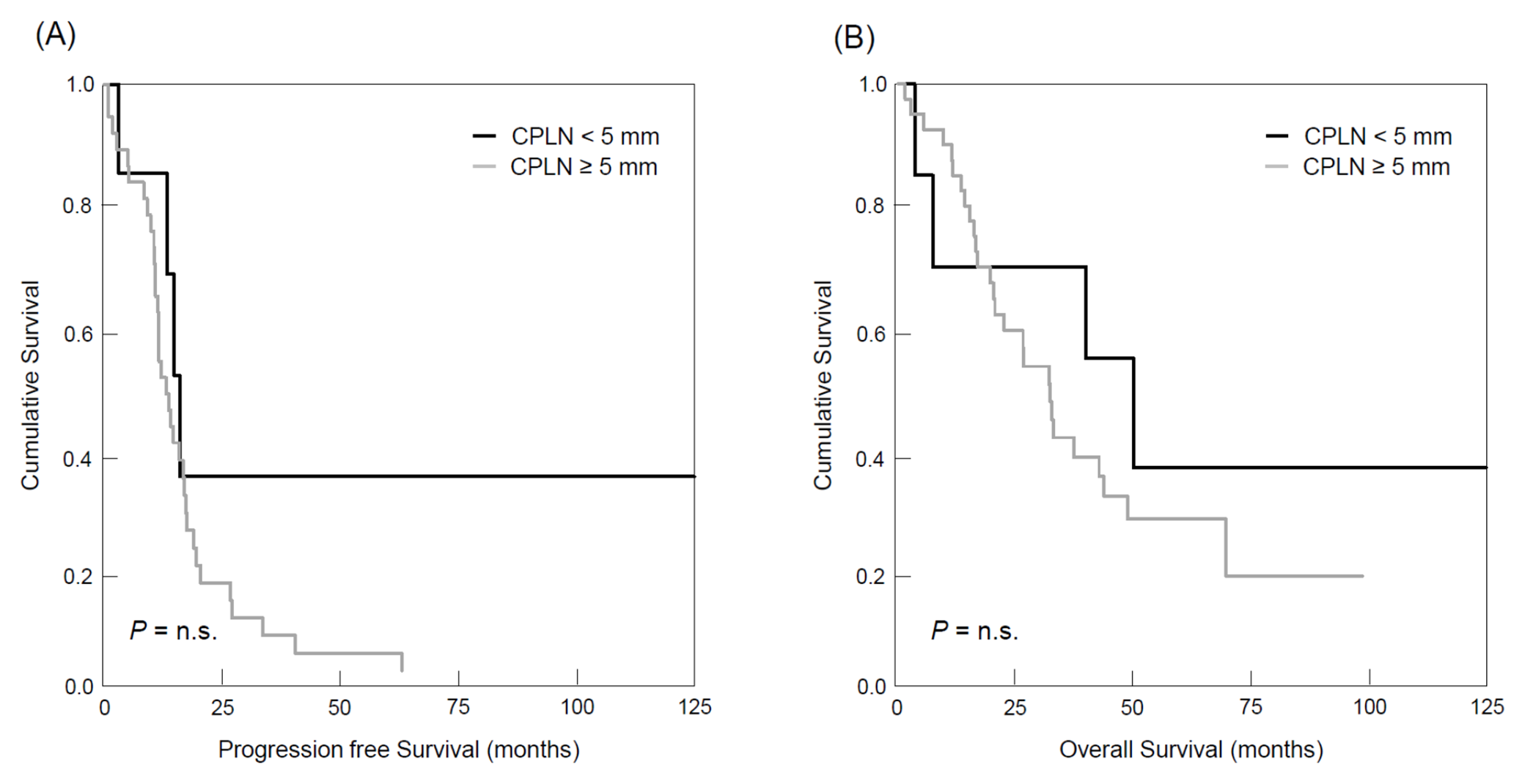

Supplement: Supplementary file 4 — Figure S4 [file AOGS-99-1092-s004.tif]
