# Supplementary material for: Enlarged cardiophrenic lymph nodes predict disease involvement of the upper abdomen and the outcome of primary surgical debulking in advanced ovarian cancer
Source: Acta Obstet Gynecol Scand. 2020 Mar 18;99(8):1092–9. doi: 10.1111/aogs.13835 (PMC7496971; doi:10.1111/aogs.13835)
Supplement: Supplementary file 5 — Table S1 [file AOGS-99-1092-s005.docx]

**TABLE S1.** Overview on published CPLN cutoffs, detection rates, and FIGO upstaging in comparison to the current series

| **Study** | **N** | **FIGO** | **Radiologic Cutoff (mm)** | **Radiologic Detection rate** | **Potential Upstaging rate** | **Histologically confirmed metastases in radiologic detected enlarged CPLN** |
| --- | --- | --- | --- | --- | --- | --- |
| Holloway et al. 1997 ^6^ | 67 | II, III | ≥ 5 | 22% | 12% |  |
| Kolev et al. 2012 ^9^ | 212 | III, IV | > 5 | 43% | 30% |  |
| Hynninen et al. 2012 ^23^ | 30 | IIC - IV | ≥ 8-28  PET positive | 33%  67% | not reported  13% |  |
| Prader et al. 2016 ^17^ | 196 | IIIC, IVA | ≥ 10 | 15% | 30% | 90% |
| Raban et al. 2015 ^16^ | 421 | II, IIIC, IV | > 10 | 11% | 9% |  |
| Garbi et al. 2017 ^18^ | 22 | IVB | ≥ 5 | not reported ^a^ | not reported ^a^ | 95% |
| Kim et al. 2016 ^22^ | 31 | III, IV | > 4  > 7 ^2^ | 100%  45% | 32%  0% | 85% |
| Salehi et al. 2017 ^19^ | 180 | IIIC, IV | > 8 | not reported ^a^ | not reported ^a^ | 83% |
| McIntosh et al. 2017 ^14^ | 88 | III | ≥ 3  ≥ 4  ≥ 5 ^b^  ≥ 6  ≥ 7  ≥ 10 | 78%  63%  43%  26%  19%  5% | 78%  63%  43%  26%  19%  5% |  |
| Mert et al. 2018 ^15^ | 253 | IIIC, IV | > 7 ^b^  > 10 | 21%  8% | 19%  8% |  |
| Prader et al. 2019 ^10^ | 350 | IIIB, IIIC, IV | ≥ 5 | 62% | 22% | 86% ^c^ |
| Current series | 178 | III, IV | ≥ 5 ^b^  ≥ 7  ≥ 10 | 50%  40%  11% | 38%  30%  6% |  |

^a^ Only patients with CPLN above the respective cutoff were included; ^b^ optimal radiologic cutoff used for all calculations in the study; ^c^ estimated in a subgroup of 52 patients, CPLN short-axis ≥ 5 mm (n = 32), ≥ 10 mm (n = 20).
